# HuR cytoplasmic expression is associated with increased cyclin A expression and poor outcome with upper urinary tract urothelial carcinoma

**DOI:** 10.1186/1471-2407-12-611

**Published:** 2012-12-21

**Authors:** Peir-In Liang, Wei-Ming Li, Yu-Hui Wang, Ting-Feng Wu, Wen-Ren Wu, Alex C Liao, Kun-Hung Shen, Yu-Ching Wei, Chung-Hsi Hsing, Yow-Ling Shiue, Hsuan-Ying Huang, Han-Ping Hsu, Li-Tzon Chen, Ching-Yih Lin, Chein Tai, Chun-Mao Lin, Chien-Feng Li

**Affiliations:** 1 Department of Pathology, Chi-Mei Foundational Medical Center, Tainan, Taiwan; 2 Department of Urology, Kaohsiung Medical University Hospital, Kaohsiung Medical University, Kaohsiung, Taiwan; 3 Institute of Biosignal Transduction, National Cheng Kung University, Tainan, Taiwan; 4 Department of Biotechnology, Southern Taiwan University of Science and Technology, Tainan, Taiwan; 5 Institute of Biomedical Science, National Sun Yat-Sen University, Kaohsiung, Taiwan; 6 Department of Urology, Chi-Mei Foundation Medical Center, Tainan, Taiwan; 7 Department of Pathology, Kaohsiung Chang Gung Memorial Hospital and Chang Gung University College of Medicine, Kaohsiung, Taiwan; 8 Department of Anesthesiology, Chi-Mei Foundation Medical Center, Tainan, Taiwan; 9 College of Medicine, China Medical University, Taichung, Taiwan; 10 National Institute of Cancer Research, National Health Research Institutes, Tainan, Taiwan; 11 Department of Internal Medicine, National Cheng Kung University Hospital, Tainan, Taiwan; 12 Institute of Molecular Medicine, National Cheng Kung University, Tainan, Taiwan; 13 Division of Gastroenterology and Hepatology, Department of Internal Medicine, Chi-Mei Foundation Medical Center, Tainan, Taiwan; 14 Department of Leisure, Recreation, and Tourism Management, Southern Taiwan University of Science and Technology, Tainan, Taiwan; 15 College of Medicine, Taipei Medical University, Taipei, Taiwan

**Keywords:** Upper urinary tract urothelial carcinoma, HuR, Cyclin A, Prognosis

## Abstract

**Background:**

HuR is an RNA-binding protein that post-transcriptionally modulates the expressions of various target genes implicated in carcinogenesis, such as *CCNA2* encoding cyclin A. No prior study attempted to evaluate the significance of HuR expression in a large cohort with upper urinary tract urothelial carcinomas (UTUCs).

**Methods:**

In total, 340 cases of primary localized UTUC without previous or concordant bladder carcinoma were selected. All of these patients received ureterectomy or radical nephroureterectomy with curative intents. Pathological slides were reviewed, and clinical findings were collected. Immunostaining for HuR and cyclin A was performed and evaluated by using H-score. The results of cytoplasmic HuR and nuclear cyclin A expressions were correlated with disease-specific survival (DSS), metastasis-free survival (MeFS), urinary bladder recurrence-free survival (UBRFS), and various clinicopathological factors.

**Results:**

HuR cytoplasmic expression was significantly related to the pT status, lymph node metastasis, a higher histological grade, the pattern of invasion, vascular and perineurial invasion, and cyclin A expression (*p* = 0.005). Importantly, HuR cytoplasmic expression was strongly associated with a worse DSS (*p* < 0.0001), MeFS (*p* < 0.0001), and UBRFS (*p* = 0.0370) in the univariate analysis, and the first two results remained independently predictive of adverse outcomes (*p* = 0.038, relative risk [RR] = 1.996 for DSS; *p* = 0.027, RR = 1.880 for MeFS). Cyclin A nuclear expression was associated with a poor DSS (*p* = 0.0035) and MeFS (*p* = 0.0015) in the univariate analysis but was not prognosticatory in the multivariate analyses. High-risk patients (pT3 or pT4 with/without nodal metastasis) with high HuR cytoplasmic expression had better DSS if adjuvant chemotherapy was performed (*p* = 0.015).

**Conclusions:**

HuR cytoplasmic expression was correlated with adverse phenotypes and cyclin A overexpression and also independently predictive of worse DSS and MeFS, suggesting its roles in tumorigenesis or carcinogenesis and potentiality as a prognostic marker of UTUC. High HuR cytoplasmic expression might identify patients more likely to be beneficial for adjuvant chemotherapy.

## Background

Urothelial carcinomas are the most common malignancy of the urinary tract and are derived from the urothelium of the upper urinary tract (renal pelvis and ureter) or lower urinary tract (urinary bladder). Upper urinary tract urothelial carcinomas (UTUCs), in contrast with urinary bladder urothelial carcinomas, are relatively rare, accounting for 2% ~ 8% of urothelial carcinomas [1]. A previous report disclosed that the ratio of incidences of urothelial carcinoma in the renal pelvis, ureter, and urinary bladder was approximately 3:1:51 [2]. However, the prevalence of UTUC is higher in Taiwan, and the ratio was 1:2.08:6.72 in a single institution study in Taiwan that included 535 cases [3]. Due to unknown reasons, the tumor stage of UTUC is high when discovered, which leads to an overall poor prognosis of patients with UTUC [4]. Currently, various prognosticatory factors have been identified, including the tumor stage, lymph node status, growth pattern, tumor necrosis, and lymphovascular invasion. Many molecular markers, such as cadherin-1, hypoxia-inducible factor (HIF)-1α, and telomerase RNA, were also found to independently be associated with tumor recurrence and poor survival [5,6].

Cyclin A is important in regulating cell cycles, including playing roles in initiating DNA replication in the S phase and preventing other cyclins from degrading. Expressions of cyclins are strictly regulated, and degradation of cyclin A in a timely manner is mandatory for the cell cycle to enter metaphase [7]. Overexpression of cyclin A and dysregulation of CDK-cyclin complexes promote tumor cell growth [8]. Cyclin A is also associated with high proliferative activity in various carcinomas, including breast cancer, lung cancer, sarcomas, and hematological malignancies [9]. Furihata et al. demonstrated that overexpression of cyclin A in UTUC is associated with poor cancer-specific survival, the tumor grade, and the tumor growth pattern [10].

HuR, a member of the embryonic lethal abnormal vision (ELAV) protein family, is a turnover- and translation-regulatory RNA-binding protein (TTR-RBP) that regulates the translation and stability of cytoplasmic messenger (m)RNA [11]. HuR was found to be upregulated in almost all malignancies tested, including carcinomas originating in the breast, colon, stomach, pancreas, esophagus, prostate, lung, thyroid, etc [12]. It binds directly to the U- and AU-rich elements in the 3’-untranslated region (UTR) of most target mRNAs, which are termed AREs, or the 5’UTR of some target mRNAs. HuR is predominantly localized in nuclei, but translocation to the cytoplasm is necessary for its regulation upon the expression of target mRNAs. HuR can stabilize many target mRNAs, including those encoding proteins that take part in tumorigenesis or carcinogenesis [13]. Furthermore, translation of several target mRNAs, including cyclin A2, can be upregulated by HuR, although the exact mechanism is still unclear.

Many studies showed that HuR is a prognostic factor in various carcinomas, such as colorectal adenocarcinoma, breast carcinoma, ovarian carcinoma, etc [14-16]. HuR stabilizes the mRNA of cyclin A2 and increases its translation. Previous studies showed that it plays a critical role in increasing the proliferative activity of colorectal carcinoma, gastric adenocarcinoma, and oral cancer [17-19]. However, correlations of HuR with biologically and clinicopathologically significant factors of UTUC are unknown.

In this study, by applying an immunohistochemical study to our well-characterized case collection, we evaluated the association of HuR overexpression with clinicopathological parameters and survival of UTUC patients.

## Methods

### Patients and tumor materials

For the immunohistochemical study and survival analysis, we retrieved data on 340 consecutive patients with primary UTUC, who had received surgical resection with curative intent (ureterectomy, *n* = 10; nephroureterectomy, *n* = 330), from the archives of Chi-Mei Medical Center (Tainan, Taiwan) between 1996 and 2004. Patients who underwent palliative resection and those with a history of previous and/or concurrent urinary bladder cancer were excluded. Patients with suspicion of lymph node metastasis received regional lymph node dissection. Cisplatin-based post-operative adjuvant chemotherapy was performed in 29 out of the 106 patients who had pT3 or pT4 disease or with nodal involvement. The criteria for the clinicopathological evaluation were essentially identical to those described in our previous work [20]. This retrospective clinical and immunohistochemical studies were approved by the institutional review board (IRB971006) of Chi-Mei Medical Center.

### Immunohistochemistry for HuR and cyclin A

After preparing and being heated for antigen retrieval as previously described, tissue sections were incubated with primary antibodies against HuR (1:100; Zymed Laboratories, South San Francisco, CA) and cyclin A (6E6, 1:50; Novocastra, Newcastle, UK) for 1 h, followed by antibody detection using a ChemMate EnVision kit (K5001; DAKO, Glostrup, Denmark). Breast carcinoma tissue with known HuR expression in the cytoplasm and cyclin A in nuclei was used as the positive control throughout. Incubation without the primary antibodies was used as the negative control.

### Interpretation and scoring of HuR and cyclin A

The immunohistochemical slides were independently interpreted by two pathologists (Y-CW and H-YH), who were blinded to the clinical and pathological results. The cytoplasmic expression of HuR and nuclear labeling of cyclin A in the UTUC were assessed using a combination of the percentage and intensity of positively stained tumor cells to generate a histological score (H-score) [21,22]. The H-score was calculated using the following equation: H-score = ∑ Pi (i + 1), where i is the intensity score (which ranged 0 ~ 4), and Pi is the percentage of stained tumor cells at each intensity (which ranged 0% ~ 100%). This formula produces a score that ranges 100 ~ 500, where 100 indicates that 100% of tumor cells were negative and 500 indicates that 100% of tumor cells were strongly stained (4+).

### Follow-up and statistical analyses

Statistical analyses were performed using the SPSS 14.0 (SPSS, Chicago, IL, USA) software package. The follow-up duration ranged 1 ~ 176 (median, 38) months. Median H-scores of cytoplasmic HuR and nuclear cyclin A were used as the cutoff to dichotomize the study cohort, separating cases into high- and low-expression groups. Associations of HuR and cyclin A expression with various clinicopathological variables were evaluated by a Chi-squared test. The association between HuR and cyclin A results was also evaluated. The end points of the analysis for the entire cohort were the disease-specific survival (DSS), metastasis-free survival (MeFS), and urinary bladder recurrence-free survival (UBRFS) which were calculated from the date of the operation on the UTUC until the presence of disease-related mortality, systemic metastasis developed, and urinary bladder recurrence occurred, respectively, or the last follow-up appointment. Univariate survival analyses were performed using Kaplan-Meier plots, and survival was evaluated by the log-rank test. In the Cox multivariate regression model, all parameters with *p* < 0.1 at the univariate level were entered to compare their independent prognostic impacts. For all analyses, two-sided tests of significance were used with *p* < 0.05 considered significant.

## Results

### Clinicopathological findings

**Figure 1:**
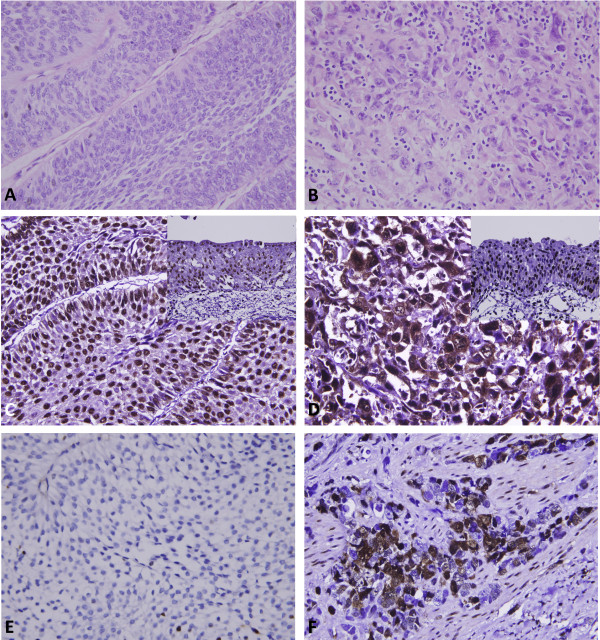
**Histology and immunohistochemistry (HuR and cyclin A) of upper urinary tract urothelial carcinomas.** Representative hematoxylin-eosin-stained sections of a low-stage urothelial carcinoma **(A)** and a high-stage, infiltrating urothelial carcinoma **(B)** which respectively demonstrated low **(C, E)** and high **(D, F)** cytoplasmic HuR and nuclear cyclin A immunoexpressions.

The clinicopathological characters of our patients are listed in Table 1. The patients’ age at diagnosis ranged 34 ~ 87 (median, 68) years. Multifocal tumors were observed in 62 cases. One hundred and forty-one cases (41.5%) had tumors involving the renal pelvis, 150 (44.1%) involving the ureter, and 49 (14.4%) involving both locations. The pT stages of 181 cases were non-invasive (Ta, Figure 1A) or early invasive (T1), and the other 159 cases were advanced stages (T2 ~ T4). The majority of cases (*n* = 284, 83%) were high-grade tumors (Figure 1B). Lymph node involvement was observed in 28 cases. Most tumors (*n* = 200) were non-invasive or had a nodular invasion pattern and demonstrated low mitotic activity (<10 per 10 high-power field, *n* = 173), while 58 and 82 cases respectively displayed a trabecular or infiltrative pattern of invasion. In addition, vascular invasion and perineurial invasion were respectively observed in 106 and 19 cases, respectively.

**Table 1:** Correlations between HuR and cyclin A expression and other important clinicopathological parameters

**Parameter**		**No. of cases**	**HuR Cyto. Exp.**^**†**^		***p*value**	**Cyclin A Exp.**		***p*value**
			**Low**	**High**		**Low**	**High**	
Gender	Male	182	93	89	0.664	85	95	0.384
	Female	158	77	81		83	75	
Age (years)	<65	138	72	66	0.508	75	63	0.185
	≥65	202	98	104		95	107	
Tumor side	Right	177	96	81	0.201	89	88	0.931
	Left	154	71	83		76	78	
	Bilateral	9	3	6		5	4	
Tumor location	Renal pelvis	141	76	65	0.374	66	75	0.607
	Ureter	150	73	77		78	72	
	Renal pelvis and ureter	49	21	28		26	23	
Multifocality	Single	278	143	135	0.261	140	138	0.779
	Multifocal	62	27	35		30	32	
Primary tumor (T)	Ta ~ T1	181	109	72	<0.001*	104	77	0.003*
	T2 ~ T4	159	61	98		66	93	
Nodal metastasis	Negative (N0)	312	166	146	<0.001*	159	153	0.237
	Positive (N1 ~ N2)	28	4	24		11	17	
Histological grade	Low grade	56	37	19	0.008*	39	17	0.001*
	High grade	284	133	151		131	151	
Pattern of invasion	Non-invasive/Nodular	200	112	88	0.030*	108	92	0.191
	Trabecular	58	24	34		27	31	
	Infiltrative	82	34	48		35	47	
Vascular invasion	Absent	234	126	108	0.035*	124	110	0.101
	Present	106	44	62		46	60	
Perineurial invasion	Absent	321	166	155	0.009*	162	159	0.479
	Present	19	4	15		8	11	
Mitotic rate (per 10 high power fields)	<10	173	89	84	0.588	103	70	<0.001*
	≥10	167	81	86		67	100	
Cyclin A expression	Low	164	95	69	0.005*	-	-	-
	High	176	75	101		-	-	

^†^ Cyto. Exp., cytoplasmic expression. * Statistically significant.

### Correlations of immunoreactivity of HuR and cyclin A with parameters in UTUC

HuR nuclear expression was detected in both normal urothelial cells and UTUCs (Figure 1C, D), but HuR cytoplasmic expression was seen in the cancer cells only. The tumors displayed a wide range of H-scores, from 100 to 480 (median, 240). After dichotomizing the tumors into low- and high-HuR expression (Figure 1C, D, respectively), as demonstrated in Table 1, high HuR expression showed a strong association with increments of the pT status (*p* < 0.001), lymph node metastasis (*p* < 0.001), a higher histological grade (*p* = 0.008), infiltrative or trabecular pattern of invasion (*p* = 0.030), vascular (*p* = 0.035) and perineurial invasion (*p* = 0.009), and cyclin A expression (*p* = 0.005).

For cyclin A nuclear expression (Figure 1E, F), H-scores ranged from 100 to 380 (median, 140). Similarly, high cyclin A expression (Figure 1F) was significantly linked to increments in the pT status (*p* = 0.003), a higher histological grade (*p* = 0.001), and frequent mitosis (*p* < 0.001).

### Survival analyses

**Figure 2:**
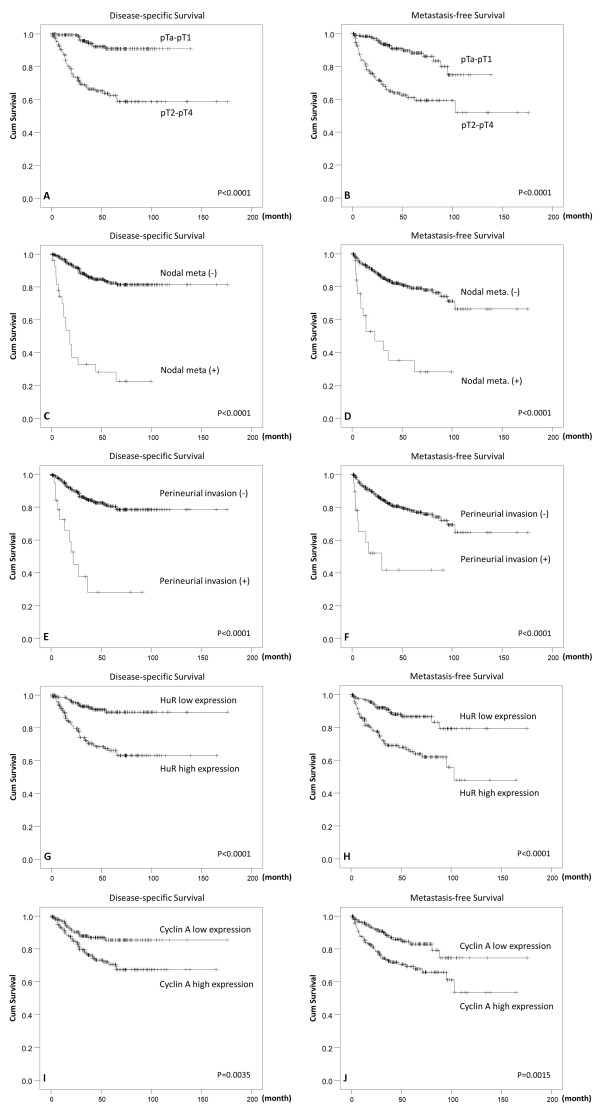
**Kaplan-Meier plots of disease-specific survival and metastasis-free survival of upper urinary tract urothelial carcinomas.** Kaplan-Meier plots show that the pT stage, nodal status, perineurial invasion, high HuR expression, and high cyclin A expression conferred significant prognostic impacts on both disease-specific survival **(A, C, E, G, I)** and metastasis-free survival **(B, D, F, H, J)**.

Associations of clinical outcomes with various clinicopathological and immunohistochemical parameters in the univariate analysis are shown in Table 2. Results showed that a poor DSS was significantly associated with the tumor location (*p* = 0.0079), multifocality (*p* = 0.0026), pT stage (*p* < 0.0001, Figure 2A), lymph node metastasis (*p* < 0.0001, Figure 2C), histological grade (*p* = 0.0215), pattern of invasion (*p* < 0.0001), vascular and perineurial invasion (both *p* < 0.0001, Figure 2E), high cytoplasmic HuR expression (*p* < 0.0001, Figure 2G), and high nuclear cyclin A expression (*p* = 0.0035, Figure 2I). All of these factors, except for the tumor location, were also strongly correlated with a worse MeFS in the univariate analysis (Table 2, Figure 2B, D, F, H, J). For UBRFS, male gender (*p* = 0.0369, Figure 2K), higher histological grade (*p* = 0.0056), and cytoplasmic HuR expression (*p* = 0.0370, Figure 2L) associated with poor outcome (Table 2).

**Table 2:** Univariate log-rank analyses for disease-specific, metastasis-free, and urinary bladder recurrence-free survival

**Parameter**		**No. of cases**	**Disease-specific survival**		**Metastasis-free survival**		**UB Recurrence-free survival**	
			**No. of events**	***p*value**	**No. of events**	***p*value**	**No. of events**	***p*value**
Gender	Male	158	28	0.8286	32	0.7904	43	0.0369*
	Female	182	33		38		36	
Age (years)	<65	138	26	0.9943	30	0.8470	34	0.5107
	≥65	202	35		40		45	
Tumor side	Right	177	34	0.7366	38	0.3074	44	0.6047
	Left	154	26		32		32	
	Bilateral	9	1		0		3	
Tumor location	Renal pelvis	141	24	0.0079*	31	0.0659	33	0.1723
	Ureter	150	22		25		32	
	Renal pelvis and ureter	49	15		14		14	
Multifocality	Single	273	48	0.0026*	52	0.0127*	64	0.1861
	Multifocal	62	18		18		15	
Primary tumor (T)	Ta ~ T1	181	11	<0.0001*	19	<0.0001*	43	0.2688
	T2 ~ T4	159	50		51		36	
Nodal metastasis	Negative (N0)	312	42	<0.0001*	55	<0.0001*	73	0.1422
	Positive (N1 ~ N2)	28	19		15		6	
Histological grade	Low grade	56	4	0.0215*	3	0.0027*	14	0.0056*
	High grade	284	57		67		65	
Pattern of invasion	Non-invasive/nodular	200	19	<0.0001*	27	<0.0001*	50	0.5398
	Trabecular	58	12		13		14	
	Infiltrative	82	30		30		15	
Vascular invasion	Absent	234	24	<0.0001*	26	<0.0001*	55	0.1770
	Present	106	37		44		24	
Perineurial invasion	Absent	321	50	<0.0001*	61	<0.0001*	75	0.2169
	Present	19	11		9		4	
Mitotic rate (per 10 high power fields)	<10	173	27	0.1607	30	0.0823	43	0.9031
	≥10	167	34		40		36	
Adjuvant chemotherapy	Not performed	311	54	0.4084	61	0.2151	70	0.2740
	Performed	29	7		9		9	
HuR cytoplasmic expression	Low	170	13	<0.0001*	19	<0.0001*	33	0.0370*
	High	170	48		51		46	
Cyclin A expression	Low	170	19	0.0035*	22	0.0015*	36	0.3784
	High	170	42		48		43	

* Statistically significant.

In the multivariate analysis, as shown in Table 3, lymph node metastasis (*p* < 0.001), perineurial invasion (*p* = 0.002), multifocality (*p* = 0.007), the pattern of invasion (*p* = 0.016), and a high histological grade (*p* = 0.026) were related to a dismal DSS. For MeFS, lymph node metastasis (*p* = 0.001), perineurial invasion (*p* = 0.025), multifocality (*p* = 0.024), a high histological grade (*p* = 0.023), and vascular invasion (*p* = 0.002) were correlated with poor outcomes. Male gender (*p* = 0.036) and high tumor grade (*p* = 0.013) significantly associated with worse UBRFS. Interestingly, high HuR expression was significantly correlated with a poor DSS (*p* = 0.038) and MeFS (*p* = 0.027) but not UBRFS (*p* = 0.150). Cyclin A expression did not associate with all three survival outcome.

**Table 3:** Multivariate analyses of disease-specific, metastasis-free, and urinary bladder recurrence-free survival

**Variable**	**Category**	**Disease-specific survival**			**Metastasis-free survival**			**UB Recurrence-free survival**		
		**Relative risk**	**95**% **Confidence interval**	***p*value**	**Relative risk**	**95**% **Confidence interval**	***p*value**	**Relative risk**	**95**% **Confidence interval**	***p*value**
Nodal metastasis	Positive vs. negative	5.602	2.912 ~ 10.776	<0.001*	3.174	1.648 ~ 6.115	0.001*	-	-	-
Perineurial invasion	Present vs. absent	3.291	1.522 ~ 7.117	0.002*	2.460	1.120 ~ 5.401	0.025*	-	-	-
Multifocality	Multifocal vs. single	2.816	1.321 ~ 6.004	0.007*	2.408	1.124 ~ 5.158	0.024*	-	-	-
Pattern of invasion	Infiltrative vs. trabecular vs. non-invasive/Nodular	1.596	1.090 ~ 2.337	0.016*	1.307	0.906 ~ 1.885	0.152	-	-	-
Histological grade	High grade vs. low grade	3.751	1.170 ~ 12.024	0.026*	4.187	1.221 ~ 14.364	0.023*	2.113	1.173-3.805	0.013*
HuR Cyto. Exp.^**†**^	High vs. low	1.996	1.039 ~ 3.834	0.038*	1.880	1.074 ~ 3.291	0.027*	1.398	0.886-2.204	0.150
Vascular invasion	Present vs. absent	1.549	0.823 ~ 2.913	0.175	2.872	1.486 ~ 5.550	0.002*	-	-	-
Cyclin A expression	High vs. low	1.632	0.909 ~ 2.932	0.101	1.703	0.994 ~ 2.918	0.053	-	-	-
Primary tumor (T)	T2 ~ T4 vs. Ta ~ T1	1.412	0.586 ~ 3.400	0.442	0.916	0.419 ~ 2.003	0.826	-	-	-
Tumor location	Both renal pelvis and ureter vs. one location alone	0.950	0.614 ~ 1.471	0.819	0.786	0.572 ~ 1.340	0.541	-	-	-
Mitotic rate	≥10 vs. <10/10 hpf	-	-	-	0.849	0.514 ~ 1.402	0.523	-	-	-
Gender	Male vs. female	-	-	-	-	-	-	1.607	1.030-2.507	0.036*

† Cyto. Exp., cytoplasmic expression. * Statistically significant.

**Figure 3:**
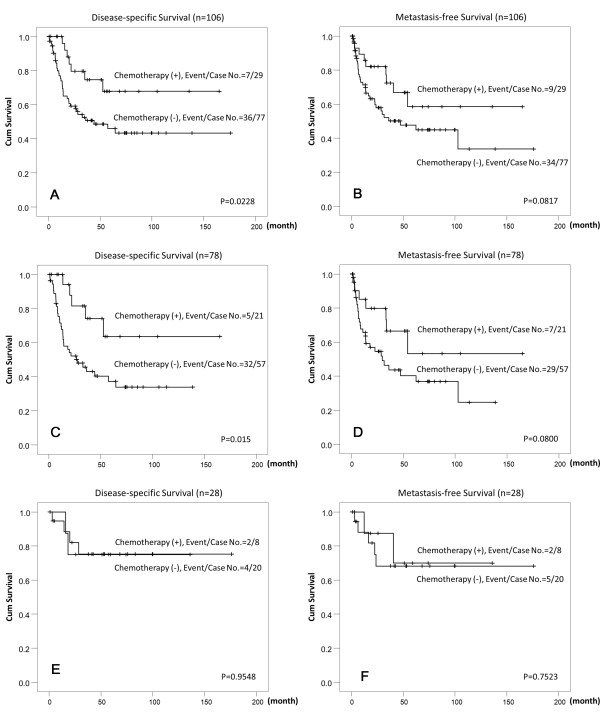
**Kaplan-Meier plots of disease-specific survival and metastasis-free survival of high-risk upper urinary tract urothelial carcinomas (pT3 or pT4 or with nodal metastasis) with or without cisplastin-based adjuvant chemotherapy.** Kaplan-Meier plots show high-risk patients who received cisplastin-based adjuvant chemotherapy conferred significant prognostic impacts on disease-specific survival (DSS) (**A**). The DSS of patients with HuR high expression in tumor cells was significantly improved by adjuvant chemotherapy (**C**). The MeFS was also improved in those with HuR high expression in tumor cells if adjuvant chemotherapy was given but was not statistically significant (**B** and **D**). Adjuvant chemotherapy did not change the DSS and MeFS in the patients with low HuR expression in tumor cells (**E** and **F**).

Adjuvant chemotherapy did not significantly improve the DSS, MeFS, and UBRFS when taking all patients into accounted (Table 2). However, the sub-group analysis for high-risk patients (pT3 or pT4 or with nodal metastasis [*n* = 106]) showed that adjuvant chemotherapy significantly improved the DSS (*p* = 0.0228, Figure 3A). Besides, high-risk patients with high HuR cytoplasmic expression (*n* = 78) had better DSS if adjuvant chemotherapy was performed (*p* = 0.015, Figure 3C). In contrast, the DSS of high-risk patients with low HuR cytoplasmic expression did not improved by adjuvant chemotherapy (*p* = 0.9548, Figure 3E). The MeFS showed a trend of improvement, in all high-risk patients (*p* = 0.0817, Figure 3B) and those with high HuR cytoplasmic overexpression (*p* =0.0800, Figure 3D), but was not statistically significant. Adjuvant chemotherapy had no effect on the MeFS of high-risk patients with low HuR patients (p = 0.7523, Figure 3F) and neither UBRFS of high-risk patients, including those with high or low HuR expression (p = 0.3178, p = 0.3870, p = 0.4054, respectively).

## Discussion

Aberrant expression of cancer-related proteins is an essential mechanism in developing malignancies. Protein manufacture can be modified through post-transcriptional mechanisms, such as mRNA splicing, transport, storage, translation, and degradation [23]. TTR-RBPs and noncoding RNA (especially microRNA) are the two main classes of factors which regulate these processes [24,25].

mRNA-binding proteins regulating various essential traits of cell biology underlying tumor aggressiveness is well established. The Hu/ELAV protein family was among the first RBPs that showed an association with carcinogenesis, after Szabo et al. discovered that HuD was a target in small-cell lung cancer-associated paraneoplastic encephalomyelitis [26]. This family is composed of one ubiquitous protein (HuR, also known as HuA) and three neuronal proteins (HuB, HuC, and HuD). As mentioned earlier, HuR is overexpressed in virtually almost all tested malignancies. It stabilizes and/or upregulates the translation of many mRNAs of cancer-related proteins. By regulating target mRNAs of these proteins, HuR expression showed the ability to enhance tumor cell proliferation, increase cell survival and local angiogenesis, evade immune recognition, and promote cancer cell invasion and metastasis [23]. In this study, we demonstrated the expression status and subcellular localization of HuR proteins in a sufficiently large cohort of UTUC cases. For those cases with immunoreactivity above the median score, HuR cytoplasmic expression was significantly correlated with poor outcomes and adverse clinicopathological factors, such as a higher histological grade, an advanced pathological status, the presence of lymph node metastasis, the pattern of invasion, and vascular/perineurial invasion. These findings suggest that HuR expression is associated with carcinogenesis of UTUC and is an important indicator of tumor aggressiveness.

Cyclin A is a crucial component in regulating the cell cycle. Cyclin A binds CDK2 when a cell enters the S-phase to stimulate DNA synthesis. Later, it binds CDK1 when a cell enters the G2 phase to initiate chromosome condensation and possibly nuclear envelope breakdown. It is degraded before a cell enters the M-phase. Overexpression of cyclin A was correlated with a poor prognosis in various malignancies, including lung cancer, breast cancer, sarcoma, and melanoma [9]. Our results show that high cyclin A expression in tumor cell nuclei was correlated with a high pT stage, a higher histological grade, and frequent mitoses. Its associations with DSS and MeFS were significant in the univariate analysis but not the multivariate analysis. These findings are comparable with previous published observations [10]. In addition, increased HuR cytoplasmic expression was correlated with high cyclin A nuclear staining, which was also compatible with what was observed in other cancers [13].

The effect of adjuvant chemotherapy in UTUC is inconclusive. Soga et al. showed that adjuvant chemotherapy with methotrexate, vinblastine, Adriamycin, and Cisplatin could prevent the intravesicle recurrence. [27] Other research groups established that there was no significant survival benefit associated with adjuvant chemotherapy. [28,29] However, our result demonstrated that adjuvant chemotherapy improved the DSS of the high-risk patients (pT3 or pT4 or with nodal involvement) in univariate analysis. Interestingly, we found that the DSS of patients with high HuR cytoplasmic expression in the tumor cells can be improved with adjuvant chemotherapy. This suggests that high HuR cytoplasmic expression might identify a subgroup of patients more likely to be beneficial by adjuvant chemotherapy. Such finding is also in line with pancreatic ductal adenocarcinoma patients. [30] Costantino et al. showed that modulation of the metabolizing enzyme of gemcitabine by HuR overexpression can enhanced the sensitivity of pancreatic cancer cells to the drug. [31] Whether such observation apply on UTUC warrant further studies.

Recently, many molecular markers that are related to cell proliferation, angiogenesis, and apoptosis were tested in UTUC tissues. Some of them proved to be prognosticatory. Snail, Bcl-2, HIF-1a, and metalloproteinases are among those that were correlated with adverse prognostic factors and poor survival [5,6]. Interestingly, mRNAs of all these markers, together with cyclin A, can be stabilized when binding to HuR [13]. In addition, the translation of mRNAs of Snail and HIF-1a is upregulated by HuR. It seems that increased expression of HuR in the cytoplasm of UTUC may stabilize and increase the production of various cancer-related proteins, and thus promote tumor aggressiveness. These may partly explain why HuR but not cyclin A was correlated with the pattern of invasion, vascular invasion, perineurial invasion, and nodal metastasis in our study.

## Conclusions

In summary, cytoplasmic HuR expression can be detected in most UTUCs but not normal urothelium, and was significantly associated with adverse clinicopathological factors. Furthermore, cytoplasmic HuR expression was positively related to cyclin A expression and can be used as an independent factor to predict poor DSS and MeFS. High HuR cytoplasmic expression might identify patients more likely to be beneficial for adjuvant chemotherapy. These results suggest that HuR may play an important role in tumorigenesis of UTUCs and confers an aggressive phenotype.

## Abbreviations

DSS: Disease-specific survival; ELAV protein: Embryonic lethal abnormal vision protein; MeFS: Metastasis-free survival; TTR-RBPs: Turnover and translation regulatory RNA-binding proteins; UBRFS: Urinary bladder recurrence-free survival; UTR: Untranslated region; UTUC: Upper urinary tract urothelial carcinoma.

## Competing interests

The authors declare that they have no competing interest.

## Authors’ contributions

W-ML, ACL, K-HS, C-HH, L-TC, and C-YL collected and reviewed the clinical information. T-FW, W-RW, Y-LS, H-PH and C-FL participated in the design of the study and provided technical support for the immunohistochemistry. P-IL, Y-CW, H-YH, and C-FL review the pathological slide, analyzed the immunohistochemistry results and interpreted the data. Y-HW, CT, and C-ML provided statistical analysis. P-IL, C-ML, and C-FL drafted the article, and all authors revised it critically for important intellectual content. All authors read and gave final approval of the version to be published.

## Pre-publication history

The pre-publication history for this paper can be accessed here:

http://www.biomedcentral.com/1471-2407/12/611/prepub

## References

[B1] Oosterlinck W Ureteral tumour: a specific upper urinary tract transitional cell carcinoma Eur Urol 2007 51 1164 1165 10.1016/j.eururo.2006.11.050 17161524

[B2] Carroll PR Tanagho EA, McAninch JW Urothelial carcinoma: cancers of the bladder, ureter and renal pelvis General Urology 1995 14 Philadelphia: Prentice-Hall 353 371

[B3] Yang MH Chen KK Yen CC Wang WS Chang YH Huang WJ Fan FS Chiou TJ Liu JH Chen PM Unusually high incidence of upper urinary tract urothelial carcinoma in Taiwan Urol 2002 59 681 687 10.1016/S0090-4295(02)01529-7 11992840

[B4] Catto JW Yates DR Rehman I Azzouzi AR Patterson J Sibony M Cussenot O Hamdy FC Behavior of urothelial carcinoma with respect to anatomical location J Urol 2007 177 1715 1720 10.1016/j.juro.2007.01.030 17437794

[B5] Chromecki TF Bensalah K Remzi M Verhoest G Cha EK Scherr DS Novara G Karakiewicz PI Shariat SF Prognostic factors for upper urinary tract urothelial carcinoma Nat Rev Urol 2011 8 440 447 10.1038/nrurol.2011.96 21727942

[B6] Lughezzani G Burger M Margulis V Matin SF Novara G Roupret M Shariat SF Wood CG Zigeuner R Prognostic factors in upper urinary tract urothelial carcinomas: a comprehensive review of the current literature Eur Urol 2012 62 100 114 10.1016/j.eururo.2012.02.030 22381168

[B7] Parry DH O'Farrell PH The schedule of destruction of three mitotic cyclins can dictate the timing of events during exit from mitosis Curr Biol 2001 11 671 683 10.1016/S0960-9822(01)00204-4 11369230 PMC2875931

[B8] Yam CH Fung TK Poon RY Cyclin A in cell cycle control and cancer Cell Mol Life Sci 2002 59 1317 1326 10.1007/s00018-002-8510-y 12363035 PMC11337442

[B9] Yasmeen A Berdel WE Serve H Müller-Tidow C E- and A-type cyclins as markers for cancer diagnosis and prognosis Expert Rev Mol Diagn 2003 3 617 633 10.1586/14737159.3.5.617 14510182

[B10] Furihata M Ohtsuki Y Sonobe H Shuin T Yamamoto A Terao N Kuwahara M Cyclin A overexpression in carcinoma of the renal pelvis and ureter including dysplasia: immunohistochemical findings in relation to prognosis Clin Cancer Res 1997 3 1399 1404 9815824

[B11] Hinman MN Lou H Diverse molecular functions of Hu proteins Cell Mol Life Sci 2008 65 3168 3181 10.1007/s00018-008-8252-6 18581050 PMC2580827

[B12] López de Silanes I Fan J Yang X Zonderman AB Potapova O Pizer ES Gorospe M Role of the RNA-binding protein HuR in colon carcinogenesis Oncogene 2003 22 7146 7154 10.1038/sj.onc.1206862 14562043

[B13] Srikantan S Gorospe M HuR function in disease Front Biosci 2012 17 189 205 10.2741/3921 22201738 PMC4540328

[B14] Yoo PS Sullivan CA Kiang S Gao W Uchio EM Chung GG Cha CH Tissue microarray analysis of 560 patients with colorectal adenocarcinoma: high expression of HuR predicts poor survival Ann Surg Oncol 2009 16 200 207 10.1245/s10434-008-0209-3 19009247

[B15] Heinonen M Bono P Narko K Chang SH Lundin J Joensuu H Furneaux H Hla T Haglund C Ristimäki A Cytoplasmic HuR expression is a prognostic factor in invasive ductal breast carcinoma Cancer Res 2005 65 2157 2161 10.1158/0008-5472.CAN-04-3765 15781626

[B16] Denkert C Weichert W Pest S Koch I Licht D Köbel M Reles A Sehouli J Dietel M Hauptmann S Overexpression of the embryonic-lethal abnormal vision-like protein HuR in ovarian carcinoma is a prognostic factor and is associated with increased cyclooxygenase 2 expression Cancer Res 2004 64 189 195 10.1158/0008-5472.CAN-03-1987 14729623

[B17] Wang W Caldwell MC Lin S Furneaux H Gorospe M HuR regulates cyclin A and cyclin B1 mRNA stability during cell proliferation EMBO J 2000 19 2340 2350 10.1093/emboj/19.10.2340 10811625 PMC384372

[B18] Mrena J Wiksten JP Kokkola A Nordling S Haglund C Ristimäki A Prognostic significance of cyclin A in gastric cancer Int J Cancer 2006 119 1897 1901 10.1002/ijc.21944 16708383

[B19] Kakuguchi W Kitamura T Kuroshima T Ishikawa M Kitagawa Y Totsuka Y Shindoh M Higashino F HuR knockdown changes the oncogenic potential of oral cancer cells Mol Cancer Res 2010 8 520 528 10.1158/1541-7786.MCR-09-0367 20332213

[B20] Huang WW Huang HY Liao AC Shiue YL Tai HL Lin CM Wang YH Lin CN Shen KH Li CF Primary urothelial carcinoma of the upper tract: important clinicopathological factors predicting bladder recurrence after surgical resection Pathol Int 2009 59 642 649 10.1111/j.1440-1827.2009.02420.x 19712132

[B21] Budwit-Novotny DA McCarty KS Cox EB Soper JT Mutch DG Creasman WT Flowers JL McCarty KS Jr Immunohistochemical analyses of estrogen receptor in endometrial adenocarcinoma using a monoclonal antibody Cancer Res 1986 46 5419 5425 3756890

[B22] McClelland RA Finlay P Walker KJ Nicholson D Robertson JF Blamey RW Nicholson RI Automated quantitation of immunocytochemically localized estrogen receptors in human breast cancer Cancer Res 1990 50 3545 3550 2187598

[B23] Abdelmohsen K Gorospe M Post-transcriptional regulation of cancer traits by HuR WIREs RNA 2010 1 214 229 10.1002/wrna.4 21935886 PMC3808850

[B24] Moore MJ From birth to death: the complex lives of eukaryotic mRNAs Science 2005 309 1514 1518 10.1126/science.1111443 16141059

[B25] Keene JD RNA regulons: coordination of post-transcriptional events Nat Rev Genet 2007 8 533 543 10.1038/nrg2111 17572691

[B26] Szabo A Dalmau J Manley G Rosenfeld M Wong E Henson J Posner JB Furneaux HM HuD, a paraneoplastic encephalomyelitis antigen, contains RNA-binding domains and is homologous to Elav and Sex-lethal Cell 1991 67 325 333 10.1016/0092-8674(91)90184-Z 1655278

[B27] Soga N Arima K Sugimura Y Adjuvant methotrexate, vinblastine, adriamycin, and cisplatin chemotherapy has potential to prevent recurrence of bladder tumors after surgical removal of upper urinary tract transitional cell carcinoma Int J Urol 2008 15 800 3 10.1111/j.1442-2042.2008.02114.x 18651862

[B28] Vassilakopoulou M de la Motte Rouge T Colin P Ouzzane A Khayat D Dimopoulos MA Papadimitriou CA Bamias A Pignot G Nouhaud FX Hurel S Guy L Bigot P Roumiguié M Rouprêt M Outcomes after adjuvant chemotherapy in the treatment of high-risk urothelial carcinoma of the upper urinary tract (UUT-UC): results from a large multicenter collaborative study Cancer 2011 117 5500 5508 10.1002/cncr.26172 21638278

[B29] Hellenthal NJ Shariat SF Margulis V Karakiewicz PI Roscigno M Bolenz C Remzi M Weizer A Zigeuner R Bensalah K Ng CK Raman JD Kikuchi E Montorsi F Oya M Wood CG Fernandez M Evans CP Koppie TM Adjuvant chemotherapy for high risk upper tract urothelial carcinoma: results from the Upper Tract Urothelial Carcinoma Collaboration J Urol 2009 182 900 906 10.1016/j.juro.2009.05.011 19616245

[B30] Richards NG Rittenhouse DW Freydin B Cozzitorto JA Grenda D Rui H Gonye G Kennedy EP Yeo CJ Brody JR Witkiewicz AK HuR status is a powerful marker for prognosis and response to gemcitabine-based chemotherapy for resected pancreatic ductal adenocarcinoma patients Ann Surg 2010 252 499 505 20739850 10.1097/SLA.0b013e3181f1fd44

[B31] Costantino CL Witkiewicz AK Kuwano Y Cozzitorto JA Kennedy EP Dasgupta A Keen JC Yeo CJ Gorospe M Brody JR The role of HuR in gemcitabine efficacy in pancreatic cancer: HuR Up-regulates the expression of the gemcitabine metabolizing enzyme deoxycytidine kinase Cancer Res 2009 69 4567 72 10.1158/0008-5472.CAN-09-0371 19487279 PMC2744447

